# The Kinase Inhibitor SFV785 Dislocates Dengue Virus Envelope Protein from the Replication Complex and Blocks Virus Assembly

**DOI:** 10.1371/journal.pone.0023246

**Published:** 2011-08-17

**Authors:** Azlinda Anwar, Takamitsu Hosoya, Kok Mun Leong, Hiroshi Onogi, Yukiko Okuno, Toshiyuki Hiramatsu, Hiroko Koyama, Masaaki Suzuki, Masatoshi Hagiwara, Mariano A. Garcia-Blanco

**Affiliations:** 1 Program in Emerging Infectious Diseases, Duke-NUS Graduate Medical School, Singapore; 2 Laboratory of Chemical Biology, Graduate School of Biomedical Science and Institute of Biomaterials and Bioengineering, Tokyo Medical and Dental University, Tokyo, Japan; 3 Laboratory of Gene Expression, Graduate School of Biomedical Science and Institute of Biomaterials and Bioengineering, Tokyo Medical and Dental University, Tokyo, Japan; 4 KinoPharma. Inc., Tokyo, Japan; 5 Division of Regeneration and Advanced Medical Science, Gifu University Graduate School of Medicine, Gifu, Japan; 6 RIKEN Center for Molecular Imaging Science, Kobe, Japan; 7 Department of Anatomy and Developmental Biology, Kyoto University Graduate School of Medicine, Kyoto, Japan; 8 Center for RNA Biology, Departments of Molecular Genetics and Microbiology, and Medicine, Duke University School of Medicine, Durham, North Carolina, United States of America; Johns Hopkins University - Bloomberg School of Public Health, United States of America

## Abstract

Dengue virus (DENV) is the etiologic agent for dengue fever, for which there is no approved vaccine or specific anti-viral drug. As a remedy for this, we explored the use of compounds that interfere with the action of required host factors and describe here the characterization of a kinase inhibitor (SFV785), which has selective effects on NTRK1 and MAPKAPK5 kinase activity, and anti-viral activity on Hepatitis C, DENV and yellow fever viruses. SFV785 inhibited DENV propagation without inhibiting DENV RNA synthesis or translation. The compound did not cause any changes in the cellular distribution of non-structural 3, a protein critical for DENV RNA synthesis, but altered the distribution of the structural envelope protein from a reticulate network to enlarged discrete vesicles, which altered the co-localization with the DENV replication complex. Ultrastructural electron microscopy analyses of DENV-infected SFV785-treated cells showed the presence of viral particles that were distinctly different from viable enveloped virions within enlarged ER cisternae. These viral particles were devoid of the dense nucleocapsid. The secretion of the viral particles was not inhibited by SFV785, however a reduction in the amount of secreted infectious virions, DENV RNA and capsid were observed. Collectively, these observations suggest that SFV785 inhibited the recruitment and assembly of the nucleocapsid in specific ER compartments during the DENV assembly process and hence the production of infectious DENV. SFV785 and derivative compounds could be useful biochemical probes to explore the DENV lifecycle and could also represent a new class of anti-virals.

## Introduction

Dengue fever, the most prevalent arthropod-borne viral diseases of humans [1], can be caused by four DENV serotypes (DENV-1, DENV-2, DENV-3, and DENV-4). DENV belongs to the *Flaviviridae* family, which comprises other medically important pathogens including the Japanese encephalitis (JEV), yellow fever (YFV) and hepatitis C (HCV) viruses [2]. There is no available effective anti-viral therapy for DENV infection and the development of a dengue vaccine is challenging because of the need to induce long-lasting protection against all four DENV serotypes simultaneously. In fact, infection with one DENV serotype does not produce lasting immunity against the other three, and during secondary infections, incomplete immunity against a new serotype can increase the likelihood of life-threatening dengue hemorrhagic fever (DHF) or dengue shock syndrome (DSS) [3]. With approximately 3.6 billion people at risk, the global distribution of all serotypes [4], and the complications inherent in vaccine development, it is essential to obtain effective therapies against DENV infection.

The DENV replication cycle begins with receptor-mediated endocytosis of the virus into cells, followed by fusion with the endosomal membrane to release the viral genome into the cytoplasm for translation and replication [2]. Replication of the viral RNA genome takes place within virus-induced endoplasmic reticulum (ER)-like vesicles, which are connected to the cytosol via pores that allow entry of factors that are required for RNA synthesis, and exit of newly synthesized viral RNA for assembly. Virus assembly occurs within ER vesicles that are in close proximity to these pores, with the ensuing accumulation of these viruses in dilated ER compartments proximal to the Golgi. All these processes occur within compartments of one ER-derived network [5], [6]. Virions are subsequently released from cells via the host cell secretory machinery. These different stages of DENV replication require complex interaction between viral and cellular factors [7], [8]. Hence, small molecules that target critical host factors could be useful for the biochemical characterization of host–virus interactions and are anti-DENV drug candidates.

In this study we describe the anti-viral activity of SFV785, a derivative of SRPIN340, a serine-arginine-rich protein kinase (SRPK) inhibitor [9]. Here we show that SFV785 inhibited the replication of HCV, and had potent anti-DENV and anti-YFV activity. While SFV785 did not inhibit the accumulation of DENV proteins and RNAs, it altered the distribution of the structural envelope (Env) protein within ER-derived vesicles, which was consistent with the altered morphology of the ER network it caused in uninfected cells. Ultrastructural electron microscopic analyses of DENV-infected SFV785-treated cells showed the presence of virion-like particles devoid of the dense nucleocapsid and thus distinctly different from viruses within enlarged ER cisternae. SFV785 did not inhibit the secretion of the virion-like particles, but inhibited the production of infectious virus. These data indicate that SFV785 inhibited the recruitment and encapsulation of the nucleocapsid in specific ER compartments during the DENV assembly process.

## Materials and Methods

This study was carried out in strict accordance with the recommendations in the Guidelines for Proper Conduct of Animal Experiments (Ministry of Education, Culture, Sports, Science, and Technology of Japan), and approved by the Tokyo Medical and Dental University (Approval number 100185).

### Cell lines and viruses

The dengue virus 2 New Guinea C strain (DENV-2) and yellow fever 17D strain (YFV) used in this study were propagated in the C6/36 (ATCC) and Vero (ATCC) cells respectively as described [10]. Baby hamster kidney (BHK-21; ATCC) and Vero cells were used for the quantification of DENV and YFV by plaque assay respectively. In brief, cells were grown in 24 well plates and infected the next day with the virus. The cells were processed for plaque forming unit (PFU) determination 6 days (DENV) or 3 days (YFV) post-infection. All statistical analyses were carried out with GraphPad Prism 4 (GraphPad Software). For the infection of control and siRNA-treated HuH-7 cells, DENV was used at a multiplicity of infection (MOI) of 1. The cells were incubated with the virus for 1 hr at 37°C with occasional rocking. After 1 hr, the cells were rinsed, overlaid with complete medium and incubated for 36 hours post-infection. For drug-treated cells, the compounds were added to the complete media after the 1 hr virus adsorption step, and incubated for the indicated amount of time. HuH-7/Rep-Feo [11] cells were maintained in DMEM and 200 µg/ml G418. HuH-7.5.1 cells [12] were maintained in DMEM supplemented with 10% fetal calf serum. The hepatitis C virus (HCV)-JFH1 was obtained from HuH-7.5-1 cells as described [13].

### Kinase screen and anti-kinase activity

All kinases were assayed at an inhibitor concentration of 10 µM and an ATP concentration of 10 µM, unless otherwise stated. Radioisotope-based assays were performed for NTRK1, SRPK1, SRPK2, CLK4 and DYRK1A as previously described [9]. The inhibition on 66 kinases was assessed by Carna Biosciences Quickscout™ Custom Profiling Service using the phosphor-peptide ELISA assays, IMAP assays and Off-chip Mobility Shift Assays (MSA). The detailed assay protocols for these kinases and assays are available from the Millipore website (http://www.carnabio.com/output/pdf/ProfilingProfilingBook_en.pdf). The inhibitory effect on the other kinases was examined with the Millipore KinaseProfiler™ Service using radioisotope-based assays. A detailed assay protocol is available from Millipore website (http://www.millipore.com/publications.nsf/a73664f9f981af8c852569b9005b4eee/5b63719614ecf1aa85257367000523ee/FILE/cd1000enus.pdf).

### Evaluation of the effect of the compounds on the replication of a HCV subreplicon

We synthesized ten derivative compounds (see Supporting Information S1) based on the chemical structure of SRPIN340 [9]. All tested compounds were dissolved in 100% DMSO, and were diluted with 100% DMSO, if necessary. Evaluation of the inhibitory effect of the compounds on the replication of HCV sub-replicon was performed as reported previously [11]. The Rep-Feo subreplicon consists of the 5′UTR of the HCV-N, the fusion gene of neomycin phosphotransferase (Neo) with the firefly luciferase (Fluc), the EMCV IRES driving translation of the NS3 to NS5B genes of HCV-N, and the 3′UTR of HCV-N. In brief, HuH-7/Rep-Feo cells were plated onto 96-well plates at 5×10^3^ cells/well 16 to 24 hrs prior to the addition of the compounds. Each compound was added at a final concentration of 10 or 20 µM in the presence of 0.04% DMSO, and incubated for an additional 48 hours. After incubation, cells were lysed with the Glo lysis buffer (Promega) and the luciferase activity measured by Bright-Glo™ Luciferase Assay System (Promega) on the ARVO MX multilabel counter (PerkinElmer Life Sciences). The luminescence intensity of each sample was reported relative to that of the well treated with 0.04% DMSO (100% luciferase activity).

### siRNA transfection procedures and analyses of gene expression

The C2 siRNA (non-specific siRNA duplex) used has been previously reported [14]. C2 and NTRK1-specific siRNAs (TrkA-3) were synthesized from Sigma, and TrkA-12 siRNA was obtained from Thermo Scientific (Table S1). siRNA transfections were performed on HuH-7 cells with using Lipofectamine 2000 as described [15]. At 78 h post-transfection, cells were plated at a density of 3×10^5^ cells per 35 mm well, and infected the day after with DENV-2 at an MOI of 1. The supernatant were harvested 36 hrs p.i., and the cells collected for plaque, western and quantitative real time PCR analyses. For Western blot analysis, harvested cells lysates were resolved on a 10% SDS-PAGE, transferred onto a nitrocellulose membrane and the appropriate proteins detected by labeling using a polyclonal anti-NTRK1 (Millipore) or anti-GAPDH (Cell Signaling Technology) antibodies. The levels of proteins were quantified using Quantity One (Bio-Rad).

### DENV-2 replicon, RNA transfection and luciferase assay

The RNA from the pDRrep DENV-2 plasmid replicon [16] was prepared as described. The replicon is shown to be able to translate and replicate similar to wild type DENV RNA, generating two peaks of luciferase activities indicative of translation from input (within first 8 hrs) and newly synthesized (48 hrs post-transfection) RNA. Briefly, the DENV-2 replicon RNA was generated from linearised pDRep by *in vitro* transcription using the RiboMax Large Scale RNA Production System (Promega). The replicon transcripts were transfected into HuH-7 cells by electroporation together with a transfection control firefly luciferase mRNA, obtained from *in vitro* transcription of a linearized pTNT-Fluc plasmid containing the firefly gene in the pTNT vector (Promega). The electroporated cells were first pooled and then aliquoted into 24-well plates. Luciferase activity, which is an indication of translation from the replicon, was measured 2 to 72 hrs post-transfection to assess for the effects of the drugs on DENV replicon activity. The analyses of both firefly and Renilla luciferase levels were performed using the dual-luciferase assay kit (Promega) on a Tecan Infinite M200 luminometer.

### RT-PCR and quantitative real-time PCR

Total RNA was prepared from infected cells using the TRIzol®Reagent (Invitrogen) according to manufacturer's instructions. The RT reaction was performed at 42°C as described [17]. All quantitative real-time PCR was performed on the iCycler iQ™ Multi-Color Real Time PCR Detection System (Bio-Rad) with the following conditions: 40 cycles of 30 s denaturation at 95°C, 30 s annealing at 55°C and 30 s extension at 72°C. Fluorescent detection of SYBR Green I (iQ SYBR Green Supermix, Bio-Rad) was carried out at the extension phase. All cDNA standards used in quantitative real-time PCR were identical in size and sequence to the targets and were generated by PCR using Taq polymerase (Promega) and 50 nM of each primer, with the following cycling conditions: a 95°C step for 3 min; 25 cycles of 95°C for 30 s, 55°C for 30 s, and 72°C for 1 min; and an elongation step at 72°C for 7 min. The quantification of viral and cellular nucleic acids in cell culture studies was normalized against the expression levels of actin. The sequences of the primers used are shown in Table S1. All statistical analyses were carried out with GraphPad Prism 4.

### Immunofluorescence

DENV-2 infected HuH-7 cells or HCV JFH-1 infected HuH 7.5-1 cells were fixed with 4% paraformaldehyde or ice-cold methanol, and processed for indirect immunoflourescence as described [15]. The proteins were labelled with anti-NS3 rabbit polyclonal (Abcam or from Dr. Padmanabhan, Georgetown University), anti-DENV-2 Env 3H5 mouse monoclonal (Chemicon), anti-calnexin rabbit monoclonal (Cell Signalling), anti-sec31 (gift from Dr. Tang B.L., NUS), anti-ERGIC-53 (Sigma-Aldrich) and anti-Giantin (Abcam) rabbit polyclonals, and visualized using secondary antibodies conjugated to FITC, rhodamine (Jackson Immunolabs) or Cy3 (Amersham). The double-stranded RNA (dsRNA), the presumed intermediate of DENV replication, was labeled with J2, a mouse monoclonal antibody (English and Scientific Consulting, Hungary). Cellular DNA was visualised with DAPI (Molecular Probes). Images were collected using a fluorescence microscope (Olympus IX700). For the quantification of JFH1-infected cells in the presence of compounds, NS3-immunostained cells were analyzed using an ArrayScan VTi. Infected cell number was defined as the number of nuclei which average signal intensity of Cy3 signal around nuclei was greater than the mean +4 S.D. of that from uninfected cells.

## Results

### Synthesis and characterization of SFV785, a kinase inhibitor that inhibits HCV replication

**Figure 1:**
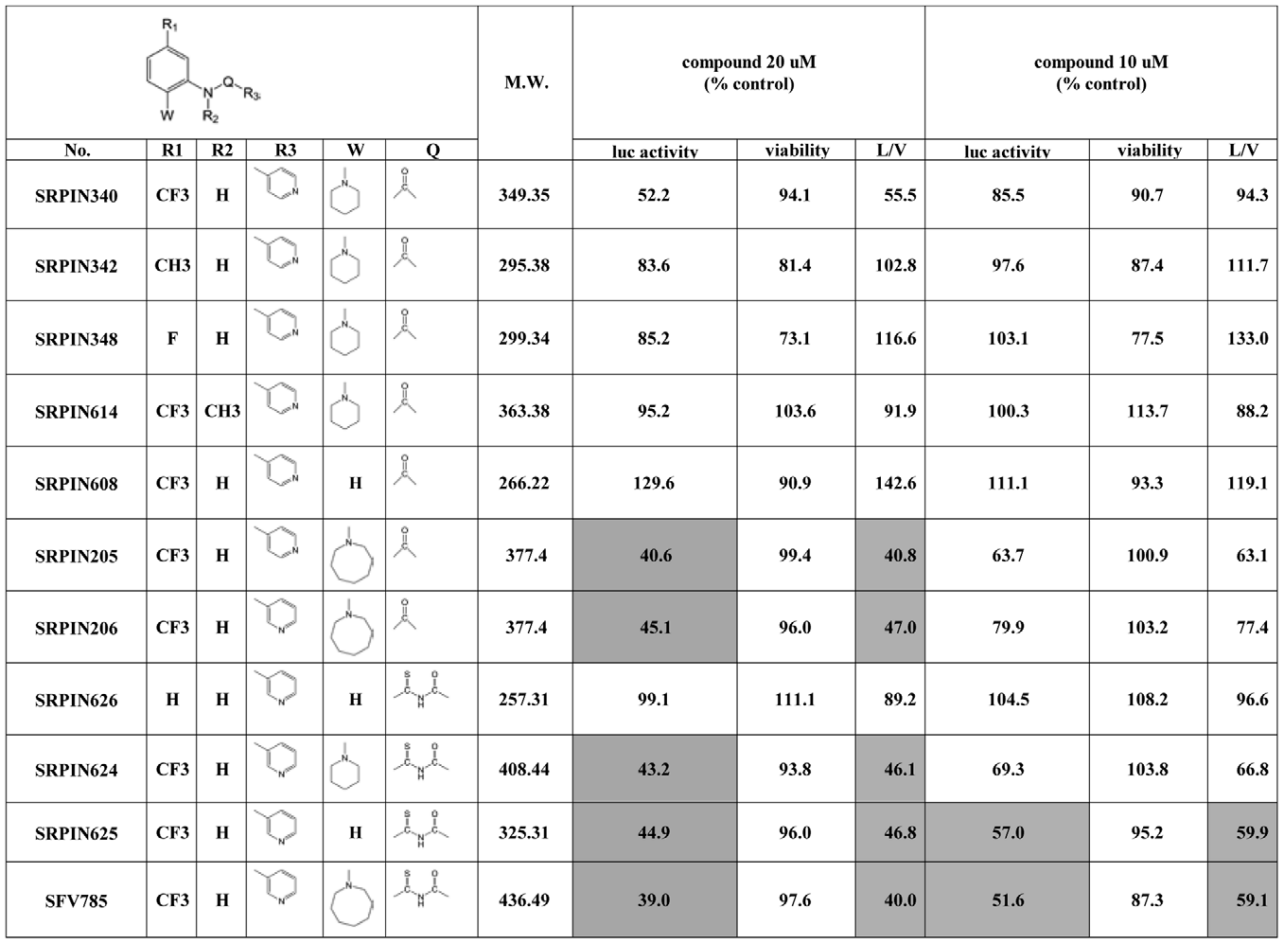
Structure-activity relationship of SRPIN340/nicotinoyl derivatives to the replication of the HCV subreplicon. The comparison of the relative luciferase activity (luc activity) and viability (viability) with the solvent (0.04% DMSO) in the presence of 10 or 20 µM of the compounds are shown. L/V is the ratio of relative luciferase activity to viability. L/V values less than 50 (20 µM) or 60 (10 µM) are highlighted. The data are represented as the mean of triplicate experiments.

SRPK inhibitor, SRPIN340, has been reported to have anti-viral activity against several RNA viruses including HIV, Sindbis virus, and HCV [9], [18]. In an attempt to optimize the anti-HCV reactivity of this inhibitor, we synthesized ten new (iso)nicotinoyl derivatives of SRPIN340 and tested them for their effects on the replication of a HCV subreplicon, Rep-Feo [11], which codes for luciferase (Figure 1). The level of luciferase activity has been previously reported to correlate with amount of HCV subreplicon RNA produced, and hence serves as a useful tool for the quantitative evaluation of the effects of compounds on HCV replication. Among the ten SRPIN340 derivatives, 1-[2-(1-azacyclooctanyl)- 5-(trifluoromethyl)] phenyl-3-nicotinoylthiourea, designated as SRPIN785 [now renamed as suppressor of flaviviridae-785 (SFV785)], had the most potent inhibitory effect on the replication of Rep-Feo (cells treated with 20 µM SFV785 had luciferase activity that was 39% of control).

**Figure 2:**
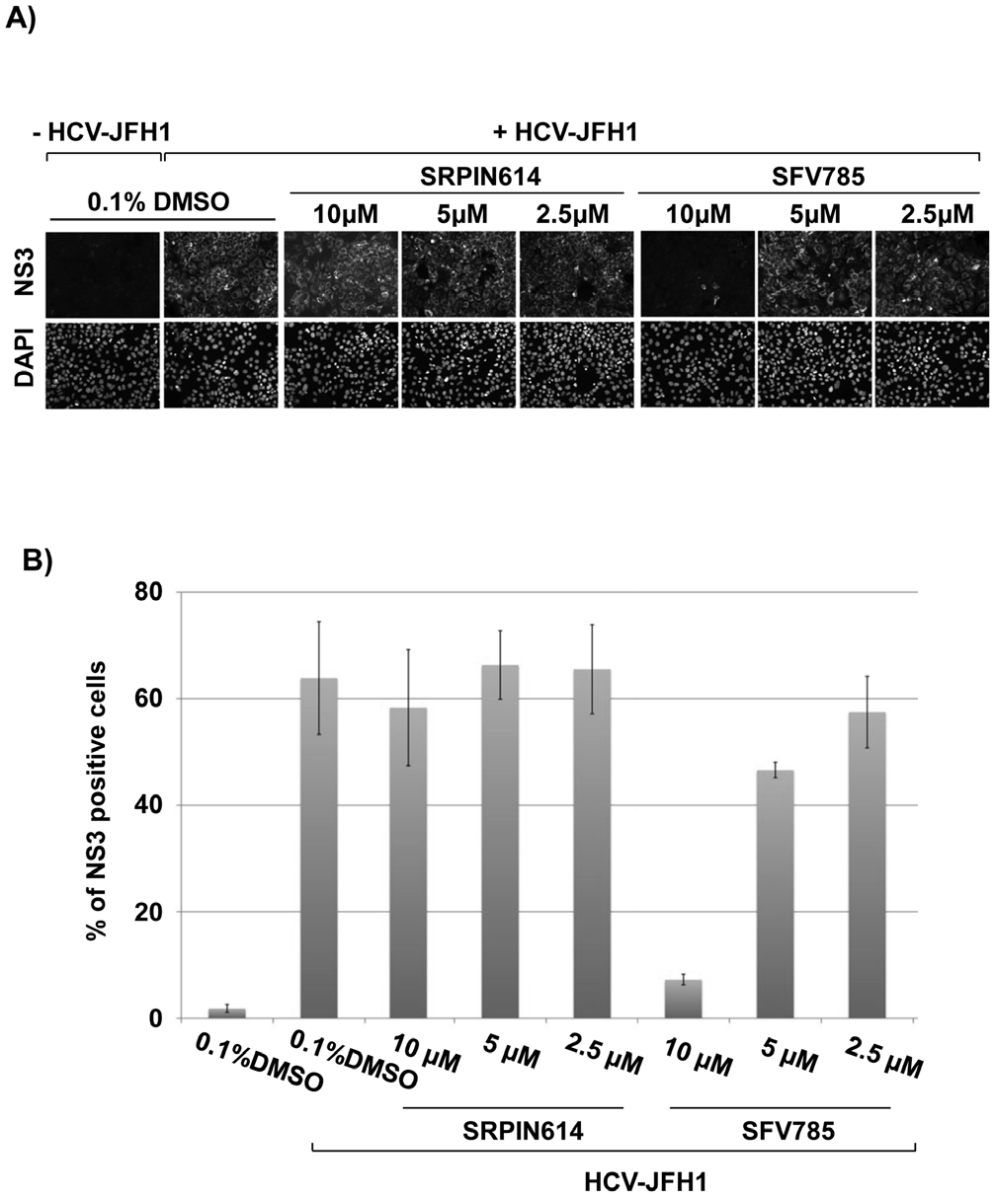
SFV785 inhibits HCV replication. (A) Detection of HCV JFH1-infected cells. HuH 7.5-1 cells were infected with HCV JFH1 and treated with the compounds SFV785 or SRPIN614 for 48 hours. The cells were fixed and immunostained with an anti-HCV NS3 antibody. (B) Quantitative analysis of HCV JFH1-infected cells. HuH-7.5-1 cells were seeded in 96 well plates, infected with HCV JFH1 and treated with the compounds SFV785 or SRPIN614 as described above. The fixed cells were immunostained with an anti-HCV NS3 mouse monoclonal antibody, and the percentage of NS3-positive cells measured using the ArrayScan VTi. The bars indicate the mean value ± S.D. from triplicate experiments.

We investigated the possibility that SFV785 could also inhibit the replication of HCV virus, JFH1. The percentage of cells infected with JFH1 at 48 hrs post-infection (p.i.) was significantly reduced in the presence 10 µM SFV785, as compared to vehicle or to a structurally similar compound, SRPIN614 (Figure 2A; Figure 1). Quantitative analysis of NS3 expression showed that about 7% of the cells were infected in the presence 10 µM SFV785, whereas about 60% of the cells were infected in the presence of SRPIN614, or vehicle (0.1% DMSO) (Figure 2B). Hence SFV785 also inhibited the replication of the intact HCV JFH1 virus, possibly more efficiently than the replication of the HCV Rep-Feo subreplicon.

The parent compound of SFV785, SRPIN340, is an inhibitor of the SR protein kinases SRPK1 and SRKP2 [18]. In order to determine if SFV785 retained its kinase inhibitory activity and specificity for SRPK, we screened the effect of this compound on the kinase activity of a library of 295 kinases *in vitro*. Kinases that showed a reduction in activity of more than 50% were considered to be potential targets for SFV785. Out of the 295 screened, SFV785 inhibited the activities of the tropomyosin receptor kinase, TrkA (NTRK1) and PRAK (MAPK-activated protein kinase 5; MAPKAPK5) to 27% and 48% respectively (Table S2), suggesting that these proteins were targets of SFV785.

### SFV785 is a potent inhibitor of DENV

**Figure 3:**
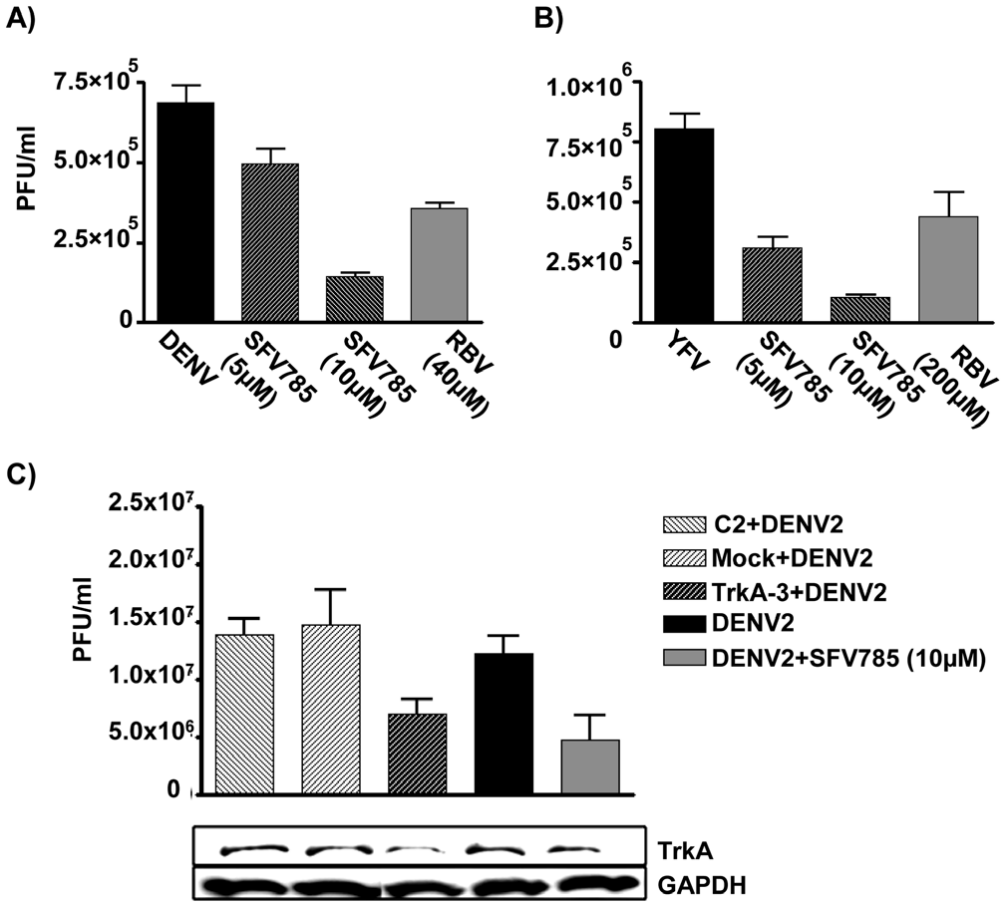
SFV785 inhibits DENV-2 and YFV propagation. (A) DENV anti-viral activity of SFV785. HuH-7 cells were infected with DENV and treated with SFV785 (5 or 10 µM) or with ribavirin (40 µM). The culture media were collected 36 hrs post-infection (p.i) and measured for viral titer by plaque assays. (B) HuH-7 cells were infected with YFV and treated with SFV785 (5 or 10 µM) or ribavirin (200 µM). The culture media were collected 12 hrs p.i and assessed by plaque assays for viral titer. The bars indicate the range of PFU/ml from three biological replicates. (C) Knockdown of NTRK1, one of the targets of SFV785, inhibited DENV propagation. The culture media and cell lysates from DENV infected HuH-7 cells treated with mock transfection, C2 or NTRK1 (TrkA-3) siRNAs, were collected 36 hrs p.i. and analysed for viral titer by plaque assays and Western blot analysis, respectively. Cells treated with SFV785 (10 µM) and infected with DENV were used as a control. The bars indicate the range of PFU/ml from three biological replicates, each in triplicates. Similar results were obtained when NTRK1 knockdown was performed using TrkA-12 siRNA.

Given the evolutionary proximity of HCV and flaviviruses, and the fact that several kinase inhibitors have been reported to inhibit flavivirus replication at different stages of their replication cycle [19], [20], [21], [22], we asked whether or not SFV785 could exhibit DENV anti-viral activity in human HuH-7 cells. SFV785 reduced the titer of DENV produced in a dose-dependent manner (Figure 3A). Ribavirin was used at a concentration of 40 µM for DENV (IC_50_) in HuH-7 cells [23]. The observed anti-viral effect of this compound was not due to cell toxicity as the maximum concentration of SFV785 used in this study (10 µM) was below its CC_50_ value (the compound concentration required to kill 50% of cells), which is >100 µM in HuH-7 cells. We tested the effect of SFV785 on YFV production and found that SFV785 treatment led to even more potent inhibition of this flavivirus (Figure 3B). DENV and YFV belong to different sero-groups within the Flavivirus genus [2]. Taken together with Figure 2, these data indicate that SFV785 has a broad-spectrum activity against *Flaviviridae*. In addition, a preliminary *in vivo* toxicity data from mice orally administered with SFV785 indicated no adverse effects or loss in body weight 7 days post-administration (Figure S1), suggesting a potential therapeutic use of this compound.

The *in vitro* kinase screens performed earlier had highlighted NTRK1 as the kinase most inhibited by SFV785. Hence we asked if silencing of endogenous NTRK1 would have any effect on DENV propagation. NTRK1-specific siRNAs (TrkA-3 or TrkA-12; Table S1) were used to reduce NTRK1 levels in HuH-7 cells. Cells infected with DENV and treated with SFV785 (10 µM) were used to compare the effects of the compound on DENV propagation (Figure 3C). The NTRK1-depleted cells were infected with DENV, the supernatants harvested at 36 hrs p.i., and subsequently titrated for infectious virus. NTRK1 knockdown resulted in a significant inhibition of infectious DENV production, validating NTRK1 as one of the *in vivo* targets of SFV785. While these data do not address how NTRK1 is involved in DENV propagation, and do not rule out yet to be identified kinases as the critical targets of SFV785, they do suggest NTRK1 as the best candidate target for the compound.

### SFV785 does not affect the intracellular accumulation of DENV gene products

**Figure 4:**
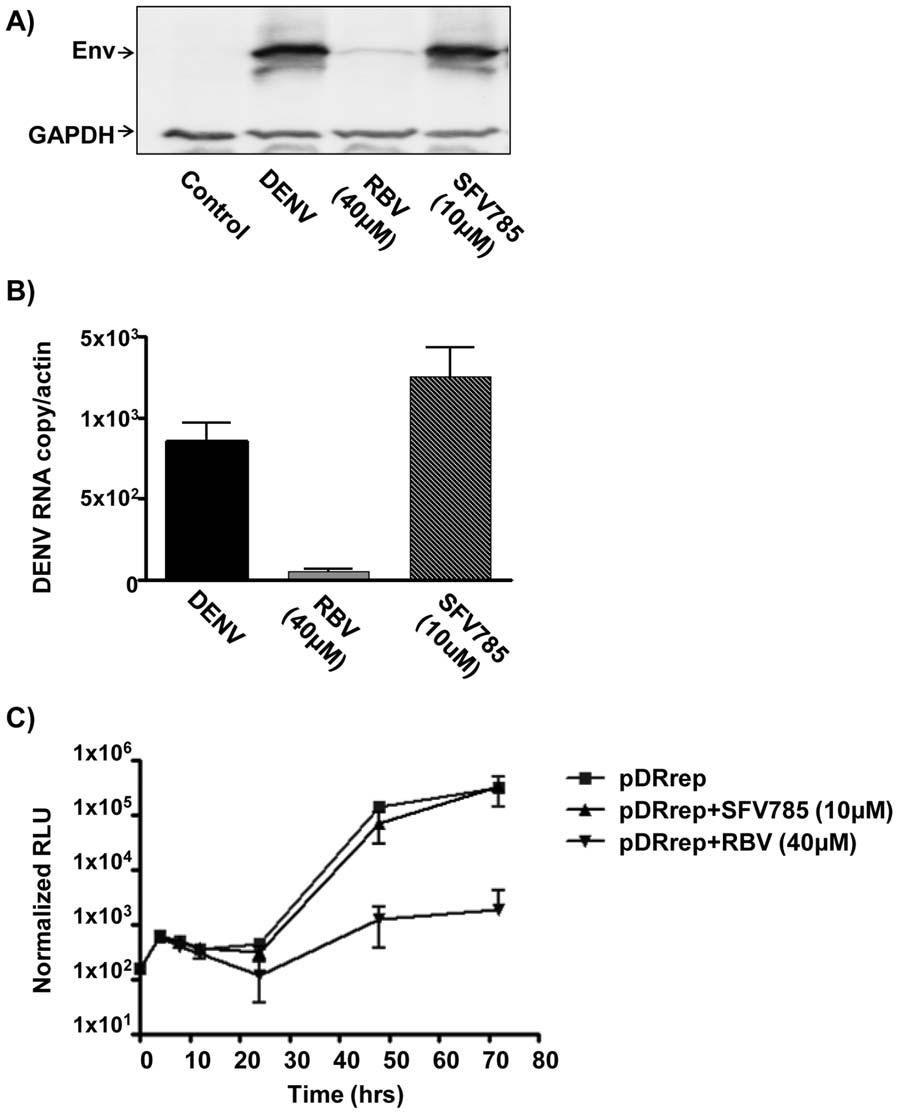
SFV785 did not inhibit DENV viral RNA synthesis or translation. HuH-7 cells were infected with DENV, and untreated or treated with SFV785 (10 µM) or with ribavirin (40 µM). The levels of (A) DENV protein in the cellular lysates by Western blot analysis, and (B) DENV RNA by real-time PCR, are shown. The bars indicate the range of DENV copy number, normalized to cellular actin, from three biological replicates, each in triplicates. (C) Effect on SFV785 on the replication of the DENV replicon. HuH-7 cells electroporated with the DENV replicons were pooled and aliquoted in media untreated or treated with SFV785 (10 µM) or ribavirin (40 µM). The cells were harvested at the indicated time points and the lysates assessed for luciferase activity. The bars indicate the S.D. from 4 separate experiments.

In order to assess the step of the viral life cycle affected by SFV785, we measured the viral protein and RNA levels. SFV785 did not inhibit viral protein or viral RNA synthesis (Figure 4A and B). These observations were reaffirmed using HuH-7 cells electroporated with the DRep DENV luciferase replicon, and grown in media with or without SFV785. Cells treated with ribavirin, a guanosine ribonucleoside analogue, were used as a control for the inhibition of viral transcription without affecting input RNA translation. SFV785 did not inhibit viral translation or RNA synthesis of the DENV replicon, with a luciferase activity profile similar to that of untreated cells (Figure 4C). As expected, cells treated with ribavirin showed inhibition of viral RNA replication but not translation. These data suggested that SFV785 did not inhibit viral RNA synthesis or translation, and indeed, intracellular accumulation of DENV gene products was unaffected.

### SFV785 dislocates the DENV envelope protein from the replication complex

**Figure 5:**
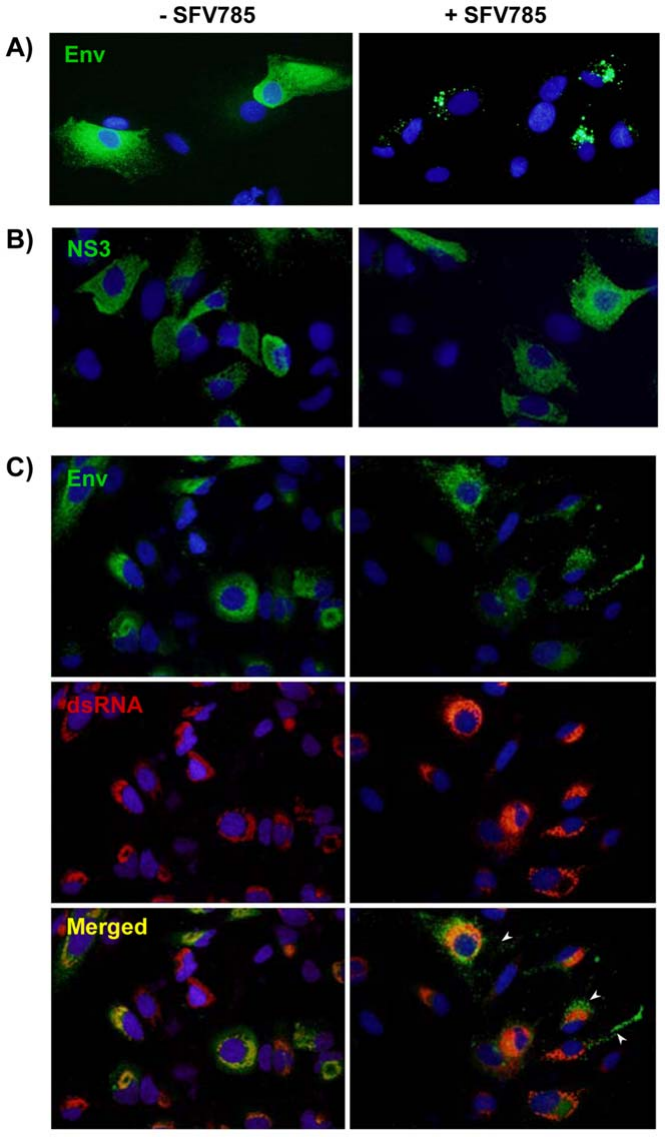
The distribution profile of the dengue viral Env, but not the NS3, is altered in infected cells treated with SFV785, and does not co-localize with the replication complex. HuH-7 cells were infected with DENV and were either untreated or treated with SFV785 (10 µM) for 36 hrs. The distribution profiles of the DENV (A) Env and (B) NS3 proteins were visualized by fluorescent microscopy. (C) DENV Env (green) and dsRNA (red) were visualized by fluorescent microscopy. Arrowheads indicate representative cells showing no co-localization of the vescicular-distributed Env with the dsRNA, a presumed marker for the DENV RNA replication. The nuclei in all experiments were stained with DAPI (blue).

We assessed the sub-cellular localization of DENV gene products by indirect immunofluorescence using antibodies to the viral Env and non-structural 3 protein (NS3) proteins. In untreated HuH-7 cells the Env protein showed the expected reticulate distribution, consistent with our earlier data [15]. In SFV785 treated cells, however, Env protein displayed discrete enlarged vesicular distribution at the periphery of the nucleus (Figure 5A). Unlike the Env protein, the pattern of NS3 in SFV785-treated DENV-2-infected cells was indistinguishable from that in the untreated DENV-2-infected cells (Figure 5B), indicating that the distinct punctuate distribution of Env was specific. We next determined the distribution of the discrete vesicles of Env with respect to the dsRNA. In untreated cells the large majority of Env signal overlapped with dsRNA, which we interpreted as tight colocalization of Env and dsRNA, a marker for the DENV replication complex [5]. In SFV785 treated cells the pattern of Env distribution was very different from the pattern observed for dsRNA and a large portion of the Env signal did not overlap the dsRNA signal (Figure 5C, arrows). This implies that SFV785 dislocated a significant portion of Env away from the replication complex.

**Figure 6:**
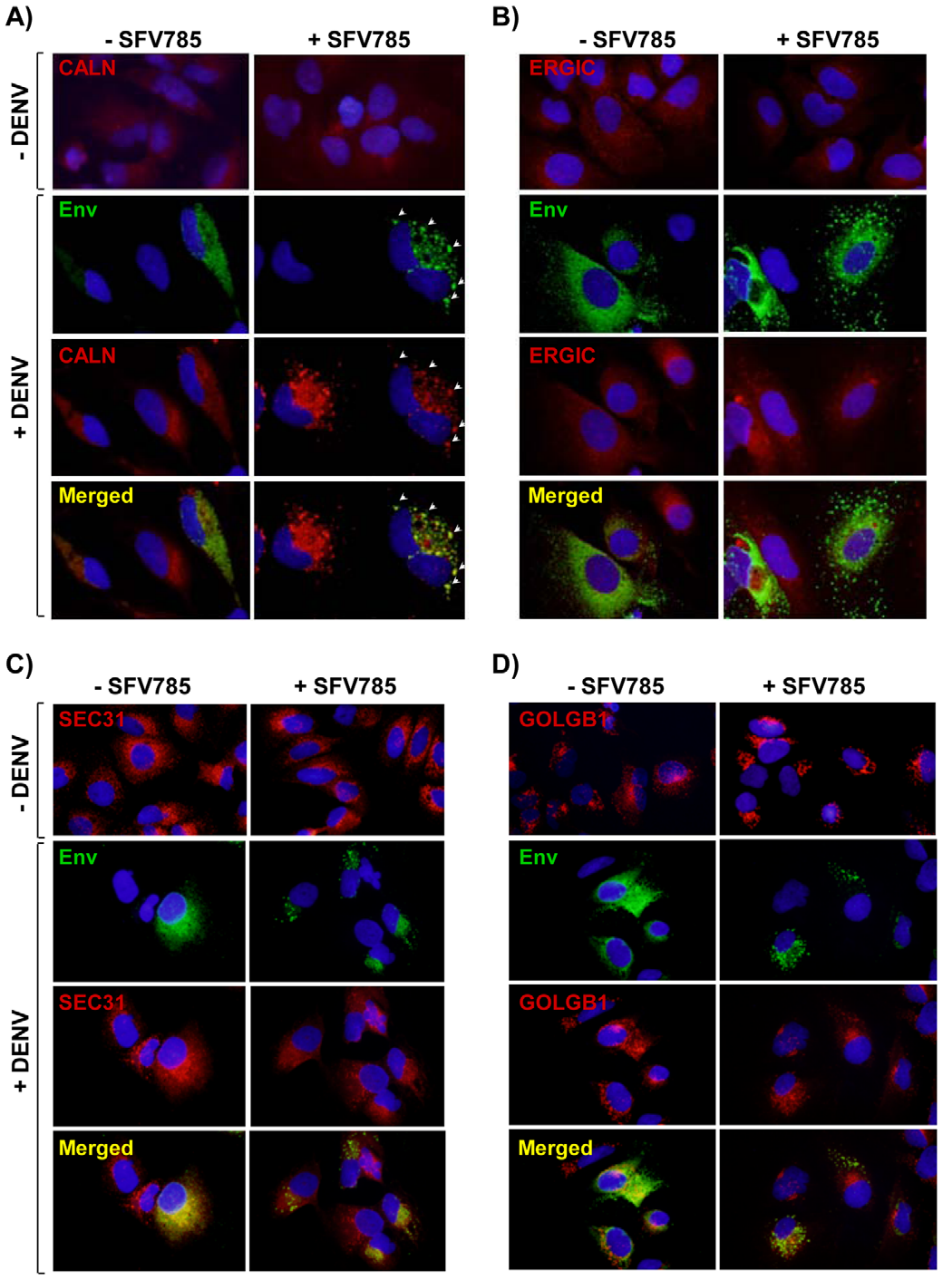
Intracellular DENV protein is located within the endoplasmic reticulum in SFV785-treated cells. The subcellular localization of DENV Env (green) proteins in DENV-infected HuH-7 cells, untreated or treated with SFV785 (10 µM), was visualized by fluorescent microscopy, with the nuclei stained with DAPI (blue). Antibodies used for the detection of cellular marker proteins are indicated in the respective panels (red). The cellular markers used were (A) calnexin (CALN) for the endoplasmic reticulum (ER), (B) ERGIC for the ER-Golgi intermediate compartments, (C) SEC31 for the COPII vesicles, and (D) GOLGB1 for the Golgi.

As the Env is one of three structural proteins of an infectious DENV, it is possible that an altered distribution of this protein may affect the production of infectious DENV. In order to determine the sub-cellular localization of the Env protein in SFV785-treated cells, we stained for cellular markers of the ER (calnexin; CALN), the ER-Golgi intermediate compartment (ERGIC), COPII vesicles (sec31) and the Golgi organelles (giantin; GOLGB1) [5]. As with our earlier results, Env in untreated cells showed a distribution profile consistent with a reticulate ER distribution, as shown by the distribution of CALN. In contrast, in SFV785 treated cells, Env was in enlarged vesicles, with the majority of the Env protein found to co-localize with the ER chaperone CALN (Figure 6A) and not with the other cellular markers (Figure 6B–D). In addition, in both uninfected and infected cells, SFV785-treatment altered the distribution profile of the CALN, but not of the other markers. It is worth noting that in this study, the level of CALN was observed to increase with DENV infection, consistent with earlier reports of a bulk up-regulation in its expression in infected and bystander cells [24], [25]. Taken together, the data suggest that SFV785 may cause morphological changes to the distribution of the specific compartments of the ER. These changes alter the sub-cellular distribution of Env, but not of NS3, suggesting a block in virion assembly.

### SFV785 alters ER structure and results in the formation of DENV viral particles with abnormal morphology

**Figure 7:**
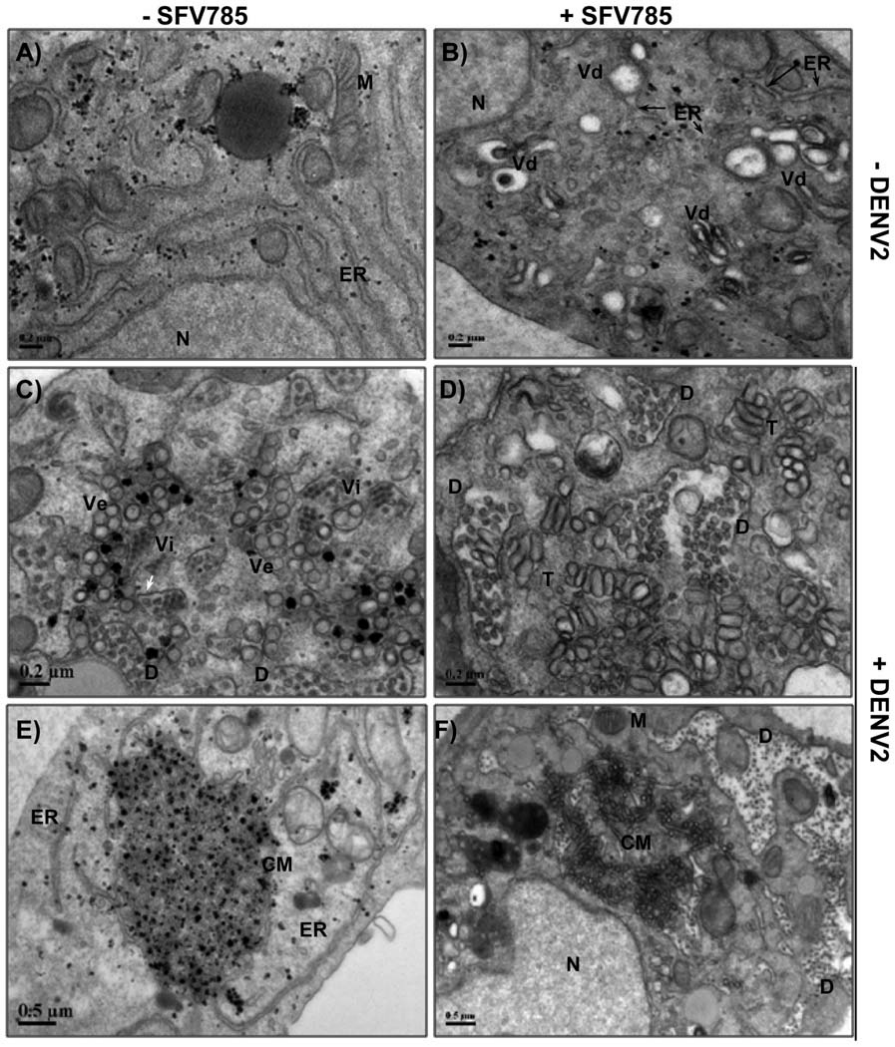
Ultrastructural analysis of DENV-infected or uninfected HuH-7 cells that were either untreated or treated with SFV785. HuH-7 cells were mock-infected (A, B) or infected with DENV (C–F) at an MOI of 1, and either untreated (A, C, E) or treated with SFV785 (10 µM) (B, D, F). Cells were fixed at the late cycle of infection, incubated with 4% paraformaldehyde-PBS solution, and processed and analyzed by electron microscopy. CM, convoluted membranes; D, probable defective virus; ER, endoplasmic reticulum; M, mitochondria; N, nucleus; T, virus-induced tubules; Vd, probable drug-induced vacuoles; Ve, virus-induced vesicles; Vi, stacked DENV particles.

The presence of DENV Env protein within ER-derived vesicles and the reduction of extracellular infectious virus, suggested that the consequence of SFV785 treatment could be a failure to form and/or release of infectious virus particles. In order to examine the effect of SFV785 on DENV propagation in detail, we used electron microscopy. HuH-7 cells were treated with 10 µM SFV785, infected with DENV at an MOI of 1 and processed for electron microscopy (EM). Enlarged vesicles (Vd) were observed in SFV785 treated uninfected HuH-7 cells, but were absent in untreated uninfected cells (Figure 7A and B). The close proximity to the ER (arrows) suggested that these drug-induced vesicles were derived from the ER. DENV-infected cells, whether untreated (Figure 7C and E) or treated (Figure 7D and F), contained virus-induced ER-derived convoluted membranes (CM), tubules (T) and vesicles (Ve) that were characteristic of flavivirus infection [5]. Stacks of enveloped virus with dense nucleocapsid core (Vi, ∼50 nm) were observed within the dilated ER cisternae in DENV-infected cells (Figure 7C). In addition, viral particles (D) that were devoid of the dense nucleocapsid core and with diameters larger than the expected for DENV were found within the ER cisternae, even in those containing stacks of virions (white arrow). The presence of defective viral particles within the cells was not surprising since the production of defective flaviviral particles has been previously reported [26], [27]. We observed a significant amount of defective viral particles (∼60 nm) in SFV785 treated DENV-infected cells, and these were in close proximity to virus-induced membranous structures (Figure 7D and F). These results suggested that SFV785 induced the formation of discrete vesicles, likely of ER origin. When administered to DENV-infected cells, SFV785 caused an abnormal morphological distortion of these vesicles, which prevented the proper assembly of the enveloped virus. The formation of these enlarged vesicles in EM was consistent with the discrete vesicles observed in the immunofluoresence studies above.

### SFV785 does not affect the secretion of virus particles

**Figure 8:**
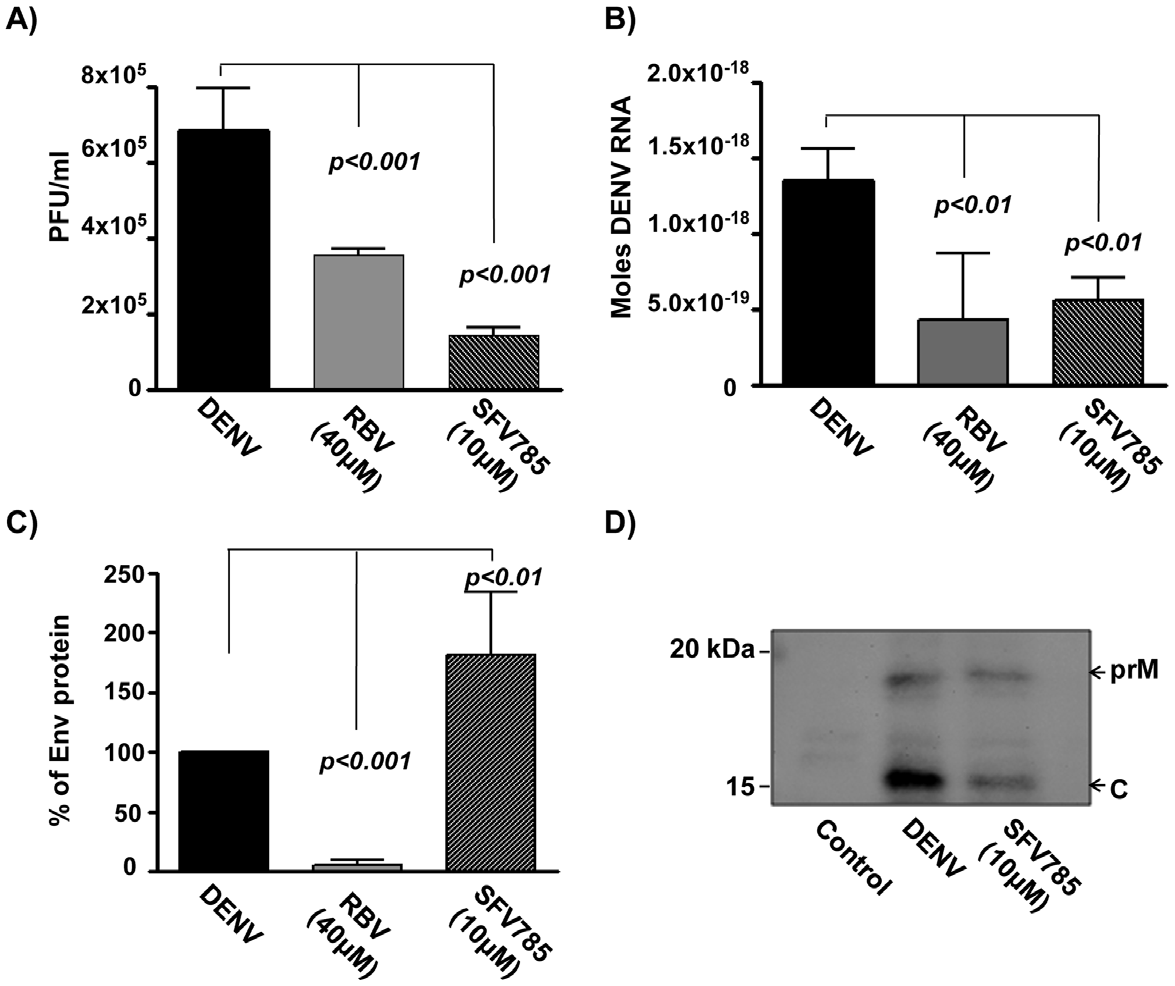
SFV785 induces the production of non-infectious virus lacking the nucleocapsid, without affecting viral secretion. HuH-7 cells were infected with DENV and either untreated or treated with SFV785 (10 µM) or with ribavirin (40 µM). The culture media were collected 36 hrs p.i., and measured for (A) the titers of infectious DENV produced by plaque assay and (B) the supernatant viral RNA. The bars indicate the S.D. from at least 3 separate experiments. (C) Comparison of the concomitant levels of DENV protein from the supernatants of treated cells in (A). The amount of DENV proteins was quantified by antigen-capture ELISA and shown as a percentage of the DENV proteins in the control untreated (DENV) supernatant. The bars indicate the range of DENV proteins from triplicates of 2 independent experiments. P values indicate significant difference by 1-way ANOVA and Tukey's post-test. (D) Western blot analysis of the capsid (C) and pre-membrane (prM) structural proteins from ultracentifugation-concentrated supernatants of HuH-7 cells that were uninfected (control) or infected with DENV-2 and treated with or without SFV785.

In order to determine if the secretion of DENV was affected by SFV785, the supernatants of treated DENV-infected cells were harvested 36 hrs p.i., and the amounts of DENV infectious particles, proteins and viral RNA determined by plaque, antigen-capture ELISA and real-time PCR assays respectively. Consistent with our previous results, treatment with SFV785 and ribavirin inhibited the production of infectious virus (Figure 8A), with a concomitant decrease in the level of viral DENV RNA in the culture supernatants (Figure 8B). As expected, the supernatants of ribavirin treated cells contained lower levels of DENV proteins. In contrast, the supernatants of SFV785-treated cells were found to have higher levels of viral Env proteins (1.8 fold) as compared to untreated cells (Figure 8C). Taken together with the data in Fig. 3, these results suggested that DENV RNA synthesis, translation, and subsequent viral particle secretion were not affected by SFV785. The higher production of defective viral particles in the culture supernatants of SFV785-treated versus untreated infected cells (Figure 8C) was somewhat surprising. Nonetheless, similar observations have been reported in cultures of JEV with a change in the capsid protein that prevents proper virus assembly [28].

EM analyses (Figure 7) and the above data suggested that the DENV particles produced in SFV785-treated cells lacked a nucleocapsid. To examine this, cell culture supernatants from SFV785 treated and DENV-2 infected 3×10^7^ HuH-7 cells were concentrated by ultracentifugation and analysed for the presence of capsid (C) and pre-membrane (prM) structural proteins. The supernatants from DENV-infected, SFV785 treated cells were observed to express ∼50% less C proteins, when normalised to the level of the prM (Figure 8D), suggesting the presence of DENV viral particles lacking capsid. Hence the results suggested that SFV785 inhibited DENV propagation by disrupting the proper assembly of the nucleocapsid into virions.

## Discussion

Protein phosphorylation by kinases is known to modulate signal transduction pathways that promote cell survival [29], [30] and immune evasion [31], [32] during DENV infection, and the regulation of endocytosis of other viruses (10). There are currently a limited number of kinase inhibitors that has been documented to inhibit DENV or flavivirus propagation at different stages of their replication cycle [19], [20], [21], [22]. Here we describe the synthesis and characterization of the anti-viral activity of a kinase inhibitor, SFV785. Our findings support the action of SFV785 at the assembly stage of infectious DENV within the ER.

The effect of SFV785 on virus assembly, and not viral RNA synthesis or translation within the ER compartment, can be placed in context by a recent study of the three-dimensional architecture and assembly sites of DENV replication [5]. The study showed that the processes of DENV RNA replication and translation occur in virus-induced vesicles within the lumen of ER, which are in close proximity to, but distinct from, ER compartments that are involved in virus assembly. These assembled viruses traverse the ER network to dilated ER cisternae proximal to the Golgi, where they accumulate as densely packed stacks before being transported into the secretory pathway [5]. Consistent with the above study, we observed the accumulation of viral particles and stacked enveloped viruses within the ER compartments in SFV785 treated cells and no inhibition in their secretion despite perturbations of the ER structure. The majority of the viral particles secreted, however, lacked the nucleocapsid, resulting in a reduction of infectious virus titers. It is interesting to note that while the cellular localization of the Env proteins was disrupted in the presence of SFV785, that of NS3 was not. DENV utilizes the viral RNA-dependent RNA polymerase (NS5), in concert with the host and other viral NS proteins (including NS3), to synthesize complementary negative-strand RNAs from the genomic RNA template, which in turn serves as templates for the synthesis of new positive-strand RNAs [2]. Currently it is not clear how the DENV RNA genome is packaged within the ER-derived prM-Env vesicles after RNA synthesis. Thus this unexpected dichotomy on the cellular localization of Env and NS3 with SFV785 can be potentially be useful in dissecting these steps in the DENV life cycle.

SFV785 was shown to be an inhibitor to the NTRK1 and MAPKAPK5 kinases. Reports of the involvement of protein phosphorylation in the assembly of flaviviruses have been limited. In HCV, the phosphorylation of the NS5A protein is critical for its interaction with the core protein for the production of infectious virus particles in HuH-7 cells [33] and for viral RNA synthesis [34]. Phosphorylation of the HCV NS2A by CK2 kinase is also reported to be necessary for the maturation step that confers infectivity on assembled particles, without affecting protein processing or genome replication [35]. In Vero cells, the c-Src tyrosine kinase is involved in the budding of the DENV nucleocapsid into the ER lumen [19], while the c-Yes kinase is required for the trafficking of the West Nile virus (WNV) through the secretory pathway [36]. NTRK1 is a tyrosine kinase and is closely involved in the diseases Hereditary Sensory and Autonomic Neuropathy (HSAN) IV and V [37], [38]. Reports on the involvement of NTRK1in virus propagation is limited, with NTRK1 reported to be critical in maintaining the latency of herpes simplex virus (HSV) in cells [39], [40]. MAPKAPK5, a major stress-activated kinase, activates the heat shock protein 27 [41], which has been implicated to be involved in the replication of various viruses, including HSV-1 [42], HCV as a NS5A interacting partner [43], and influenza virus as one of the host proteins detected in the virus particle [44]. It is formally possible that SFV785 inhibits other yet to be identified proteins that act at multiple steps of the DENV replication cycle, leading to improper assembly of infectious DENV.

Our studies indicate that SFV785 impinges on the DENV life cycle by exerting its effect on the ER network itself. The dynamics of the ER network is maintained by a plethora of proteins including reticulons and cytoskeletal proteins [45]. NTRK1 is reported to be involved in signalling pathways that modulate cellular or ER membrane dynamics at specific sites via the reorganization of the cytoskeletal proteins [46], [47], [48]. The capsid (C) of HCV, a basic protein that is responsible for genome packaging, is reported to interact with the α- and β-tubulin for efficient infection and to promote virus transport and/or assembly in infected cells [49], [50]. In HCV and DENV infected cells, the accumulation of cytoplasmic viral C proteins on the surface of lipid droplets, which are ER-derived organelles [51] , is thought to be crucial for the formation of infectious viruses [52], [53]. Hence perturbations of assembly-associated ER compartments by SFV785 may interfere with the recruitment of the nucleocapsid with the structural proteins, thus reducing the production of infectious viruses

SFV785 is a broad-spectrum *Flaviviridae* inhibitor and it is likely that it targets a common pathway required for the replication of these viruses. Our toxicity data indicated that SFV785 was well-tolerated when fed to mice at a dose of 1 g/kg/day for 1 week. Hence the current studies not only indicate the usefulness of SFV785 as a tool for the biochemical characterization of critical host–virus interactions, but also underscore the potential of the compound as a chemical starting point for anti-DENV pharmacological agents.

## Supporting Information

Figure S1 **SFV785 did not affect the growth of mice.** The growth and body weight of ICR-mice were monitored pre- and post-administration of SFV785 (1 g/kg/day) or vehicle alone at day 0. No significant weight differences were detected in mice with or without SFV785 administration. Error bars indicate the range of weight (n = 6).
(TIF)

Table S1 **Sequences of primers and siRNAs used in the study.** Sense and antisense primers were denoted with suffixes ‘f’ and ‘r’ respectively. The primers were designed according to sequences reported in Genbank accession numbers M29095 (DENV-2) and NM001101 (actin).
(PDF)

Table S2 **Kinase profiling of the inhibitory effect of SFV785.** The inhibitory effect of SFV785 was tested on 295 kinases. Kinases which showed a reduction of more than 50% in activity with 10 µM of SFV785 were shaded in grey. The HUGO nomenclatures of TrkA (NTRK1) and PRAK (MAPKAPK5) are within the parentheses.
(PDF)

Supporting Information S1 **Supporting Methods and Materials.**
(DOCX)
