# Supplementary figures and images for: The Kinase Inhibitor SFV785 Dislocates Dengue Virus Envelope Protein from the Replication Complex and Blocks Virus Assembly
Source: PLoS One. 2011 Aug 17;6(8):e23246. doi: 10.1371/journal.pone.0023246 (PMC3157368; doi:10.1371/journal.pone.0023246)

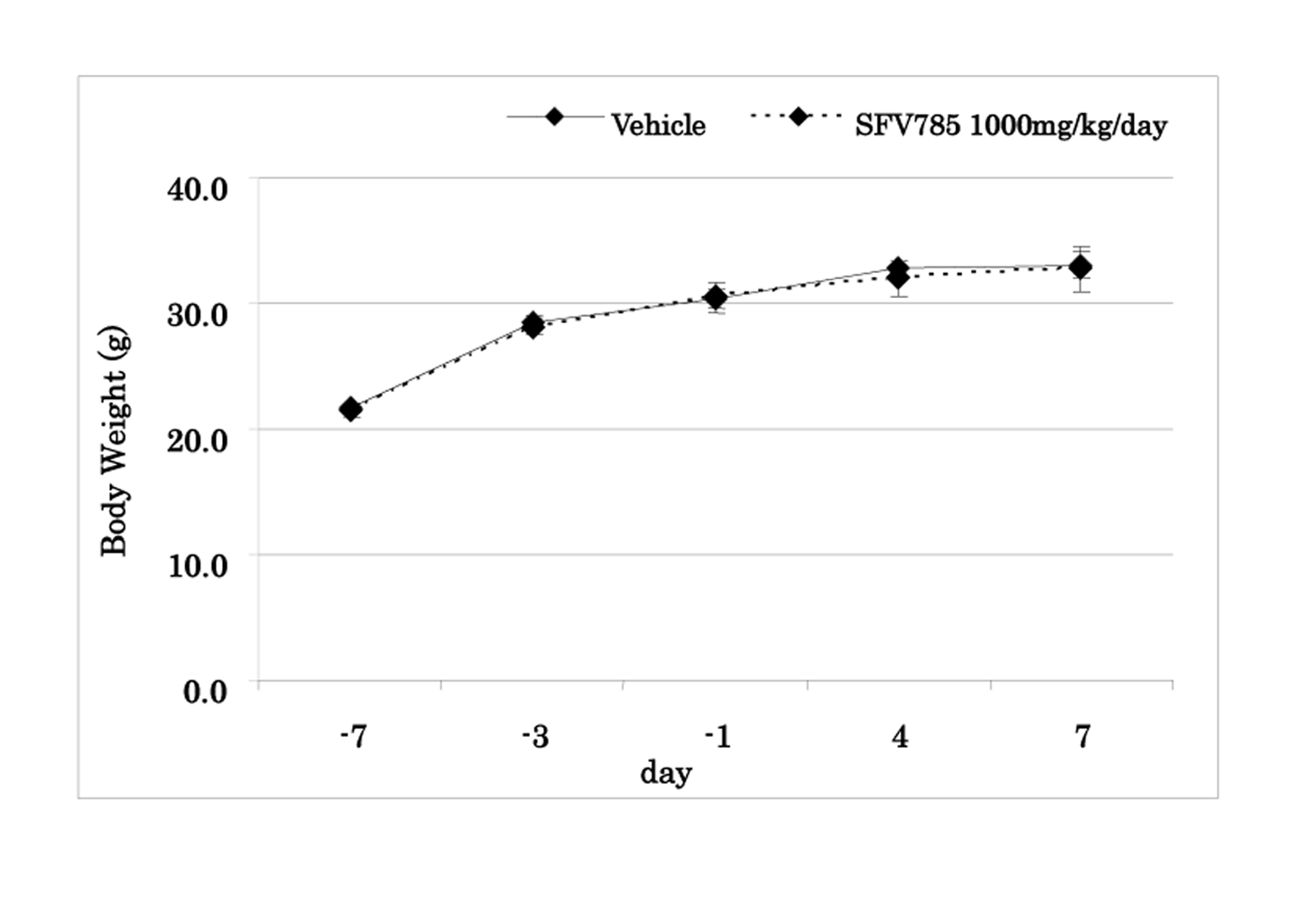

Supplement: Figure S1 — SFV785 did not affect the growth of mice. The growth and body weight of ICR-mice were monitored pre- and post-administration of SFV785 (1 g/kg/day) or vehicle alone at day 0. No significant weight differences were detected in mice with or without SFV785 administration. Error bars indicate the range of weight (n = 6). (TIF) [file pone.0023246.s001.tif]
