# Supplementary material for: The Kinase Inhibitor SFV785 Dislocates Dengue Virus Envelope Protein from the Replication Complex and Blocks Virus Assembly
Source: PLoS One. 2011 Aug 17;6(8):e23246. doi: 10.1371/journal.pone.0023246 (PMC3157368; doi:10.1371/journal.pone.0023246)
Supplement: Table S1 — Sequences of primers and siRNAs used in the study. Sense and antisense primers were denoted with suffixes ‘f’ and ‘r’ respectively. The primers were designed according to sequences reported in Genbank accession numbers M29095 (DENV-2) and NM001101 (actin). (PDF) [file pone.0023246.s002.pdf]

Table S1: Sequences of primers and siRNAs used in the study.

	Sequence	Position
Real-time primers		
DENV-f	5’ ACCTGGGAAGAGTGATGGTTATGG 3’	3632-3655
DENV-r	5’ ATGGTCTCTGGTATGGTGCTCTGG3’	3813-3836
Actin-f	5’ GAGCACAGAGCCTCGCCTTT 3’	15-34
Actin-r	5’ TGACCCATGCCCACCATCAC 3’	200-219
	**Target Sequence**	**Gene Accession no.**
siRNA		
TrkA-3	5’ GUGACGUGCUGGGCAGAGA 3’	NM_001007792
TrkA-12	5’ CAACAAAUGUGGACGGAGA 3’	NM_001007792

Sense and antisense primers were denoted with suffixes ‘f’ and ‘r’ respectively. The primers were designed according to sequences reported in Genbank accession numbers M29095 (DENV-2) and NM001101 (actin).
