# Supplementary material for: The Kinase Inhibitor SFV785 Dislocates Dengue Virus Envelope Protein from the Replication Complex and Blocks Virus Assembly
Source: PLoS One. 2011 Aug 17;6(8):e23246. doi: 10.1371/journal.pone.0023246 (PMC3157368; doi:10.1371/journal.pone.0023246)
Supplement: Table S2 — Kinase profiling of the inhibitory effect of SFV785. The inhibitory effect of SFV785 was tested on 295 kinases. Kinases which showed a reduction of more than 50% in activity with 10 µM of SFV785 were shaded in grey. The HUGO nomenclatures of TrkA (NTRK1) and PRAK (MAPKAPK5) are within the parentheses. (PDF) [file pone.0023246.s003.pdf]

Table S2: Kinase profiling of the inhibitory effect of SFV785

No.	Enzyme	Activity (%)	No.	Enzyme	Activity (%)	No.	Enzyme	Activity (%)	No.	Enzyme	Activity (%)	No.	Enzyme	Activity (%)	No.	Enzyme	Activity (%)
1	Abl	101	51	CSK	106	101	GSK3β	91	151	MGC42105	101	201	PHKG1	95	251	SAPK3	118
2	ACK1	100	52	c-RAF	100	102	Haspin	81	152	MINK	98	202	PHKG2	99	252	SAPK4	109
3	ALK	104	53	cSRC	121	103	HER2	94	153	MKK4	112	203	PhKγ2	83	253	SGK	124
4	ALK4	115	54	CTK	81	104	HER4	96	154	MKK6	118	204	Pim-1	123	254	SGK2	89
5	Arg	115	55	DAPK1	78	105	Hck	106	155	MKK7β	102	205	Pim-2	107	255	SGK3	120
6	AMPK	109	56	DAPK2	93	106	HGK	97	156	MLCK	94	206	Pim-3	85	256	SIK	121
7	Arg	119	57	DCAMKL2	184	107	HIPK1	97	157	MLK1	98	207	PKA	95	257	SLK	102
8	ARK5	95	58	DDR1	98	108	HIPK2	97	158	MLK2	111	208	PKBα	93	258	Snk	100
9	ASK1	102	59	DDR2	110	109	HIPK3	98	159	MLK3	99	209	PKBβ	133	259	SPHK1	98
10	Aurora-A	85	60	DLK	109	110	HIPK4	96	160	MNK1	108	210	PKBγ	87	260	Src(1-530)	117
11	Aurora-B	101	61	DMPK	113	111	IGF-1R	92	161	Mnk2	100	211	PKCα	92	261	SRM	102
12	Aurora-C	94	62	DRAK1	124	112	IKKα	165	162	MOS	96	212	PKCβI	101	262	SRPK1	84
13	Axl	97	63	DYRK1B	95	113	IKKβ	119	163	MRCKα	103	213	PKCβII	90	263	SRPK2	95
14	Blk	70	64	DYRK2	92	114	IKKe	101	164	MRCKβ	123	214	PKCγ	89	264	STK33	105
15	Bmx	133	65	DYRK3	98	115	IR	103	165	MSK1	117	215	PKCδ	95	265	Syk	71
16	BMPR1A	103	66	eEF-2K	115	116	IRR	95	166	MSK2	105	216	PKCε	94	266	TAK1	90
17	BRAF	95	67	EGFR	98	117	IRAK1	101	167	MSSK1	133	217	PKCη	90	267	TAO1	100
18	BRK	91	68	EphA1	89	118	IRAK4	102	168	MST1	96	218	PKCι	93	268	TAO2	84
19	BrSK1	103	69	EphA2	101	119	Itk	109	169	MST2	95	219	PKCμ	97	269	TAO3	94
20	BrSK2	112	70	EphA3	103	120	JAK2	106	170	MST3	109	220	PKCθ	132	270	TBK1	98
21	BTK	102	71	EphA4	118	121	JAK3	91	171	MST4	86	221	PKCζ	109	271	TEC	86
22	CaMKI	100	72	EphA5	103	122	JNK1α1	110	172	mTOR	83	222	PKD1	124	272	Tie2	87
23	CaMK2a	92	73	EPHA6	98	123	JNK2α2	92	173	MuSK	100	223	PKD2	96	273	TLK2	106
24	CaMKIIβ	84	74	EphA7	105	124	JNK3	97	174	NDR1	105	224	PKD3	101	274	TNK1	101
25	CaMKIIγ	92	75	EphA8	103	125	KDR	92	175	NEK1	105	225	PKG1α	116	275	TRKA (NTRK1)	27
26	CaMKIδ	122	76	EphB1	141	126	Lck	115	176	NEK2	97	226	PKG1β	114	276	TRKB	112
27	CaMKIIδ	104	77	EphB2	106	127	LIMK1	119	177	NEK3	88	227	PKN1	103	277	TRKC	99
28	CaMKIV	121	78	EphB3	111	128	LKB1	130	178	NEK4	103	228	PKR	90	278	TSSK1	96
29	CDK1/cyclinB	106	79	EphB4	112	129	LOK	114	179	NEK6	110	229	Plk1	92	279	TSSK2	103
30	CDK2/cyclinA	91	80	ErbB4	108	130	LTK	103	180	NEK7	105	230	Plk3	99	280	TTK	91
31	CDK2/cyclinE	106	81	Erk5	62	131	Lyn	111	181	NEK9	99	231	PLK4	90	281	Txk	113
32	CDK3/cyclinE	107	82	FAK	98	132	MAPK1	83	182	NEK11	94	232	PRAK (MAPKAPK5)	48	282	TYK2	98
33	CDK5/p25	93	83	Fer	90	133	MAPK2	112	183	NLK	105	233	PRK2	105	283	ULK2	93
34	CDK5/p35	121	84	Fes	147	134	MAP2K2	83	184	p70S6K	111	234	PrKX	106	284	ULK3	110
35	CDK6/cyclinD3	108	85	FGFR1	96	135	MAP2K3	91	185	PAK1	103	235	PTK5	109	285	WEE1	124
36	CDK7/cyclinH/MAT1	121	86	FGFR2	102	136	MAP2K5	91	186	PAK2	92	236	Pyk2	113	286	WNK1	84
37	CDK9/cyclin T1	117	87	FGFR3	111	137	MAP3K1	105	187	PAK3	95	237	QIK	104	287	WNK2	99
38	CHK1	133	88	FGFR4	138	138	MAP3K2	95	188	PAK4	99	238	Ret	102	288	WNK3	108
39	CHK2	93	89	Fgr	108	139	MAP3K3	101	189	PAK5	103	239	RIPK2	107	289	VRK2	102
40	CK1γ1	91	90	Flt1	96	140	MAP3K4	90	190	PAK6	104	240	ROCK-I	103	290	Yes	102
41	CK1γ2	88	91	Flt3	63	141	MAPKAP-K2	108	191	PAR-1Bα	96	241	ROCK-II	98	291	ZAP-70	137
42	CK1γ3	92	92	Flt4	93	142	MAPKAP-K3	103	192	PASK	62	242	Ron	110	292	ZIPK	85
43	CK1δ	67	93	Fms	82	143	MARK2	100	193	PBK	104	243	Ros	105	293	PI3 Kinase (p110b/p85a)	95
44	CK2	94	94	FRK	101	144	MARK3	95	194	PDGFRα	107	244	Rse	182	294	PI3 Kinase (p120g)	69
45	CK2α2	94	95	Fyn	113	145	MARK4	95	195	PDGFRβ	114	245	Rsk1	136	295	PI3 Kinase (p110d/p85a)	80
46	CLK2	82	96	GCK	100	146	MEK1	92	196	PDK1	148	246	Rsk2	120			
47	CLK3	94	97	GRK5	105	147	MARK1	97	197	PDHK2	87	247	Rsk3	119			
48	cKit	93	98	GRK6	97	148	MELK	102	198	PDHK4	99	248	Rsk4	124			
49	COT	64	99	GRK7	107	149	Mer	93	199	PEK	95	249	SAPK2a	98			
50	CRIK	106	100	GSK3α	91	150	Met	89	200	PGK	104	250	SAPK2b	103			

The inhibitory effect of SFV785 was tested on 295 kinases. Kinases which showed a reduction of more than 50% in activity with 10 μM of SFV785 were shaded in grey. The HUGO nomenclatures of TrkA (NTRK1) and PRAK (MAPKAPK5) are within the parentheses.
