# Supplementary material for: The Kinase Inhibitor SFV785 Dislocates Dengue Virus Envelope Protein from the Replication Complex and Blocks Virus Assembly
Source: PLoS One. 2011 Aug 17;6(8):e23246. doi: 10.1371/journal.pone.0023246 (PMC3157368; doi:10.1371/journal.pone.0023246)
Supplement: Supporting Information S1 — Supporting Methods and Materials. (DOCX) [file pone.0023246.s004.docx]

SUPPORTING INFORMATION

Supporting Methods and Materials

Compound synthesis

All small molecule inhibitors used in this study were prepared according to the method described earlier (9) or the representative protocol described for the synthesis of SFV785 (1-[2-(1-azacyclooctanyl)-5-(trifluoromethyl)] phenyl-3-nicotinoylthiourea) shown below.


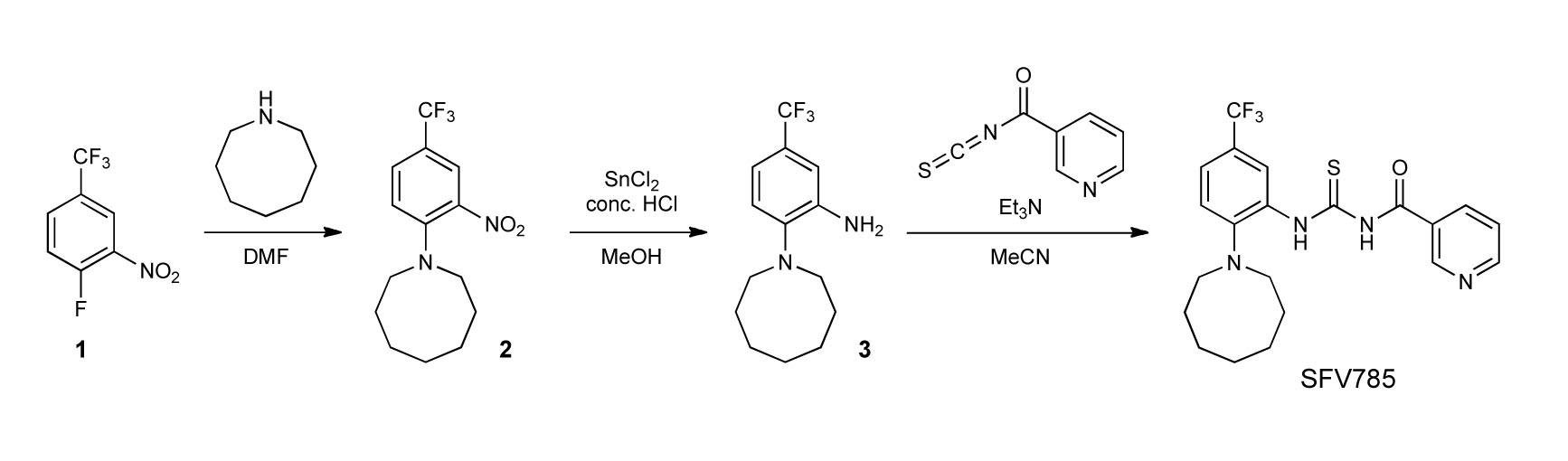


To a solution of 4-fluoro-3-nitrobenzotrifluoride (1) (550 mg, 2.63 mmol) in N,N-dimethylformamide (DMF) (4 mL) was added heptamethyleneimine (760 µL, 5.98 mmol) at 0^o^C. The mixture was allowed to warm to room temperature and stirred for 1 h. To the mixture was added water and extracted three times with ethyl acetate. The combined organic extracts was washed with brine, dried on anhydrous sodium sulfate, filtered, and then concentrated under reduced pressure. The residue was purified by silica-gel column chromatography (*n*-hexane/ethyl acetate = 10/1) to give 4-(1-azacyclooctanyl)-3-nitrobenzotrifluoride (2) (794 mg, 99.9%) as an orange oil; ^1^H NMR (CDCl_3_, 400 MHz) δ 1.53 (m, 6H, 3CH2), 1.77 (m, 4H, 2CH2), 3.40 (t, 4H, *J* = 5.8 Hz, 2CH2), 7.14 (d, 1H, *J* = 9.0 Hz, aromatic), 7.54 (d, 1H, *J* = 9.0 Hz, aromatic), 7.90 (s, 1H, aromatic).

To a solution of 4-(1-azacyclooctanyl)-3-nitrobenzotrifluoride (2) (500 mg, 1.65 mmol) in methanol (6 mL) were successively added concentrated hydrochloric acid (890 µL, 10.7 mmol) and anhydrous tin (II) chloride (1.10 g, 5.80 mmol) at 0^o^C. The mixture was allowed to warm to room temperature and stirred for 1 h. To this was added a saturated aqueous solution of sodium bicarbonate until the mixture became neutral. After filtration, the mixture was extracted three times with ethyl acetate. The combined organic extracts was washed with brine, dried on anhydrous sodium sulfate, filtered, and then concentrated under reduced pressure. The residue was purified by silica-gel column chromatography (*n*-hexane/ethyl acetate = 15/1) to give 2-(1-azacyclooctanyl)-5-(trifluoromethyl)aniline (3) (323 mg, 71.7%) as an orange oil; ^1^H NMR (CDCl_3_, 400 MHz) δ 1.69-1.80 (br s, 10H, 5CH2), 2.98-3.10 (br s, 4H, 2CH2), 4.21 (s, 2H, NH2), 6.94-6.96 (m, 2H, aromatic), 7.12 (d, 1H, *J* = 8.3 Hz, aromatic).

To a suspension of potassium thiocyanate (97.2 mg, 1.00 mmol) in acetonitrile (15 mL) was added nicotinoyl chloride hydrochloride (356 mg, 2.00 mmol) at room temperature and the mixture was heated at 70^o^C for 40 min. After cooling the mixture to room temperature, to this were successively added a solution of 2-(1-azacyclooctanyl)-5-(trifluoromethyl)aniline (3) (272 mg, 999 µmol) in acetonitrile (5 mL) and triethylamine (278 µL, 2.00 mmol) at room temperature and then stirred with heating at 50^o^C for 1 h. After cooling the mixture to room temperature, to this was added water and the mixture was extracted three times with ethyl acetate. The combined organic extracts was washed with brine, dried on anhydrous sodium sulfate, filtered, and then concentrated under reduced pressure. The residue was purified by silica-gel column chromatography (n-hexane/ethyl acetate = 2/1) to give 1-[2-(1-azacyclooctanyl)-5-(trifluoromethyl)]phenyl-3-nicotinoylthiourea (SFV785) (405 mg, 92.9%) as a yellow solid; Melting point; 102-105^o^C; ^1^H NMR (CDCl_3_, 400 MHz) δ 1.65-1.76 (m, 10H, 5CH2), 2.98-3.10 (m, 4H, 2CH2), 7.22 (d, 1H, *J* = 8.8 Hz, aromatic), 7.44 (d, 1H, *J* = 8.4 Hz, aromatic), 7.50-7.53 (m, 1H, aromatic), 8.21 (m, 1H, aromatic), 8.28 (s, 1H, aromatic), 8.89 (d, 1H, *J* = 4.8 Hz, aromatic), 9.11 (s, 1H, NH), 9.17 (d, 1H, *J* = 2.4 Hz, aromatic), 12.1 (s, 1H, NH).

Cell viability in the presence of the compounds

The viable cell ratio HuH-7/Rep-Feo cells after 48 hours treatment by compounds was measured using Cell Count Reagent SF (NACALAI TESQUE), according to the manufacturer’s instruction. Absorbance at 450nm was measured using an ARVO MX multilabel counter (PerkinElmer Life Sciences). For background correction, same set of compound-DMEM mixture without HuH-7/Rep-Feo cells were prepared and test as above. The relative cell viability was calculated as: Relative Viability (%) = (a-m) / (p-n) x 100 where a: was absorbance at 450 nm obtained from sample well; p: value from the well with 0.04% DMSO; m: value from the well with compound but without HuH-7/Rep-Feo cells, and n: value from the well with 0.04% DMSO but without HuH-7/Rep-Feo cells.

Concentration of viral supernatants and Western blot analysis

HuH-7 cells (3x10^7^ cells) were grown in T175 cm^2^ flasks and were infected with DENV-2 at an MOI of 1, and treated with or without SFV785 for 48 hours. The supernatants were harvested and cleared by centrifugation at 4000 rpm for 30 mins. The clarified supernatants were then concentrated by ultracentrifugation at 45000 rpm for 2 hrs. The pellets were then resuspended in 250 µl of phosphate-buffered saline (PBS) and the resuspended viral particles analysed for structural proteins by Western blot analysis using DENV2-C2 anti-capsid (a gift from Dr. Subhash Vasudevan, Duke-NUS) and 2H2 anti-prM (a gift from Dr. Sheemei Lok, Duke-NUS) monoclonal antibodies. The detected bands were quantified using Quantity One 1-D analysis software (Bio-Rad).

Electron microscopy

DENV-2 infected and mock-infected HuH-7 cells were infected with DENV-2 for 40 hrs and processed for LR white resin embedding. In brief, the cells were first fixed with 4 % paraformaldehyde in PBS, pH 7.4. The fixatives were removed by a series of washing with PBS. The cells were then scraped and centrifuged at 3000 rpm for 10 mins, processed for dehydration with increasing concentration of ethanol. The dehydrated cells were further infiltrated with LR White resin and embedded into BEEM capsules. The embedded cells are then cut and ultrathin sections of the infected cells were picked onto formvar-coated grids, embedded in 2% uranyl acetate for 5 min and dried before viewing under the EM208 transmission electron microscope (Philips).

ELISA assay

Antigen capture ELISA assay was performed on supernatants of infected cells as described [53] using rabbit polyclonal (USBio) and 3H5 mouse monoclonal (Chemicon) antibodies against DENV. Purified DENV-2 Env protein (a gift from Dr. P. Kukkaro, Duke-NUS) was used as a standard protein in the ELISA assay. The data were analyzed by 1-way ANOVA and Tukey’s post-test using GraphPad Prism 4.

Drug administration in mice

The body weight of two groups of 5-weeks old ICR mice (6 mice/ group) were monitored one week prior to drug administration. At day 0, the mice were orally administered with either SFV785 (1g/kg/dy) or vehicle alone, and the body weight and mortality monitored for the subsequent 7 days.

Supporting References

53. Gray, A (1999) Virus Culture: A Practical Approach. In: Cann, AJ, editor. Oxford

University Press. pp. 81-109.
